# Unlocking the Potential of the ANN Optimization in Sweet Potato Varieties Drying Processes

**DOI:** 10.3390/foods13010134

**Published:** 2023-12-29

**Authors:** Olja Šovljanski, Biljana Lončar, Lato Pezo, Anja Saveljić, Ana Tomić, Sara Brunet, Vladimir Filipović, Jelena Filipović, Jasna Čanadanović-Brunet, Gordana Ćetković, Vanja Travičić

**Affiliations:** 1 Faculty of Technology Novi Sad, University of Novi Sad, Bulevar Cara Lazara 1, 21000 Novi Sad, Serbia; oljasovljanski@uns.ac.rs (O.Š.); cbiljana@uns.ac.rs (B.L.); anav@uns.ac.rs (A.T.); sara.brunet99@gmail.com (S.B.); vladaf@uns.ac.rs (V.F.); jasnab@uns.ac.rs (J.Č.-B.); gcetkovic@uns.ac.rs (G.Ć.); vanjaseregelj@uns.ac.rs (V.T.); 2 Engineering Department, Institute of General and Physical Chemistry, Studentski trg 12/V, 11000 Belgrade, Serbia; 3 Institute of Food Technology, University of Novi Sad, Bulevar Cara Lazara 1, 21000 Novi Sad, Serbia; jelena.filipovic@fins.uns.ac.rs

**Keywords:** sweet potato, lyophilization, convective drying, osmotic dehydration, artificial neural network modeling, multi-objective optimization

## Abstract

This study explores the unexploited potential of Artificial Neural Network (ANN) optimization techniques in enhancing different drying methods and their influence on the characteristics of various sweet potato varieties. Focusing on the intricate interplay between drying methods and the unique characteristics of white, pink, orange, and purple sweet potatoes, the presented experimental study indicates the impact of ANN-driven optimization on food-related characteristics such as color, phenols content, biological activities (antioxidant, antimicrobial, anti-hyperglycemic, and anti-inflammatory), chemical, and mineral contents. The results unveil significant variations in drying method efficacy across different sweet potato types, underscoring the need for tailored optimization strategies. Specifically, purple sweet potatoes emerge as robust carriers of phenolic compounds, showcasing superior antioxidant activities. Furthermore, this study reveals the optimized parameters of dried sweet potato, such as total phenols content of 1677.76 mg/100 g and anti-inflammatory activity of 8.93%, anti-hyperglycemic activity of 24.42%. The upgraded antioxidant capability is presented through DPPH^●^, ABTS^●+^, RP, and SoA assays with values of 1500.56, 10,083.37, 3130.81, and 22,753.97 μg TE/100 g, respectively. Additionally, the moisture content in the lyophilized sample reached a minimum of 2.97%, holding favorable chemical and mineral contents. The utilization of ANN optimization proves instrumental in interpreting complex interactions and unlocking efficiencies in sweet potato drying processes, thereby contributing valuable insights to food science and technology.

## 1. Introduction

Although it originates from the South American tropics, sweet potato (*Ipomoea batatas*) is currently produced around the world with an average production of 131 million tons per year and is seventh among all food crops. The reason behind this trend lies in the high adaptability of this crop to produce a large volume of food per hectare and flexible plantation and harvest periods [1,2]. Additionally, -it presents a nutritional asset to the food industry with high nutritional value, a short growing season, tolerance to high-temperature soils with low fertility, and no significant pest or disease problems [1]. In particular, sweet potato root starch, with unique physicochemical characteristics, is well appreciated as an active food ingredient. Furthermore, roots are a good source of minerals, dietary fibers, and antioxidants, containing chemical constituents that allow them to be used to prevent and treat a wide range of diseases [2]. With the potential of use in daily diets for humans, some biological activities of sweet potatoes, such as antioxidative, hepatoprotective, anti-cancer, antidiabetic, and anti-inflammatory activity, can be selected [2,3]. However, chemical compositions and health effects must be conducted for enhanced use and multiple applications [3].

One of the main visual differences between sweet potatoes is flesh color, which also serves to determine the different varieties of this crop. Flesh varies in color from yellow, orange, red, brown, pink, and purple to beige and white. The sweetness and moisture of white or pale-yellow fleshed potatoes are noticeably lower than those of those with red, pink, or orange flesh [4]. Sweet potatoes’ biological activities result from their unique chemical composition. Their phytonutrients profile presents valuable weapons against health risks caused by free radicals [2]. For example, -yellow and orange-fleshed sweet potatoes present a unique combination of phenolic acids and a relatively high level of carotenoids. Orange-fleshed sweet potatoes are considered nature’s supreme source of β-carotene and rich in vitamin A.

Moreover, purple-fleshed sweet potatoes have high levels of acylated anthocyanins and other phenolics with antioxidant and anti-inflammatory activities. Aromatic acylated glycosyl groups in the anthocyanin of purple sweet potatoes cause rather a high pH tolerance and thermostability. Moreover, the high concentrations of carotenoids and anthocyanins in orange and purple varieties make them a perfect natural substitution for synthetic food coloring agents [5].

Besides their abundance of bioactive compounds, sweet potatoes have a high moisture level (above 80%), making them highly perishable. As storage techniques needed for preserving fresh vegetable products require costly conditions of low temperatures, drying vegetables presents an alternative solution for preserving their nutritional value, enhancing their storage stability, minimizing packaging requirements, and reducing transport weight [6]. Numerous drying techniques are used for drying fruits and vegetables, allowing storage for extended periods and reducing postharvest losses [7]. Considering these states, there is a valid assumption that some of these drying techniques can be employed for the dehydration of *I. batatas*, further using prepared dried products as functional food or its components. Among these techniques, freeze drying or lyophilization is extremely valued for the preparation of high-quality food products. In this process, water as ice is removed from a substance by sublimation, which takes place with very little pressure. Unfortunately, the lyophilization process demands a high amount of energy compared to convective drying. One of the more recent and popular dehydration methods includes osmotic dehydration, where energy is not used to generate latent heat from evaporation, reducing food moisture content by up to 50%. It is based on applying the hypertonic solution, using the concentration difference for water diffusion from the food matrix to the present solution [8]. The list of possible applications of the drying process of vegetables is very long, and most of them can be used at the industrial level; therefore, optimization of this part of vegetable processing is essential.

Dehydration/drying processes of vegetables are often time-consuming, require intensive energy consumption, and, when it comes to evaluation of effects, can be expensive, resulting in numerous unorganized and complex data. Therefore, some advanced mathematical tools can be used to define and understand the characteristics of dried samples quickly and effectively. One of them, which also can uncover complex relationships in data, is artificial neural networks (ANNs) [9]. There is limited research on the manipulation of vegetable drying conditions using previously trained ANNs [10] and no available literature on the application of ANNs for predicting phytochemical characteristics and biological activities of different *I. batatas* varieties with different drying conditions. ANN models are acknowledged as proficient modeling tools because they can furnish empirical solutions for problems derived from experimental datasets. These models exhibit aptitude in managing intricate systems characterized by nonlinearities and interactions among decision variables [11,12].

This research aimed to employ the influence of different drying methods on the presence of bioactive components, biological activities, potential, chemical and mineral compositions, and colors of different sweet potato varieties using advanced mathematical optimization design. To unlock the potential of multi-objective ANN optimization, several continuous steps in experimental and data analysis are conducted:

1. Sample preparation;
2. Drying process;
3. Comprehensive characterization (color, chemical and mineral composition, phenolic content, antioxidant capacity, pharmaceutical activities, and antimicrobial potential);
4. Mathematical analysis (principal component, correlation, cluster analysis, and descriptive statistics);
5. Artificial neural network modeling;
6. Optimization of parameters of dried sweet potato.

## 2. Materials and Methods

**Figure 1:**
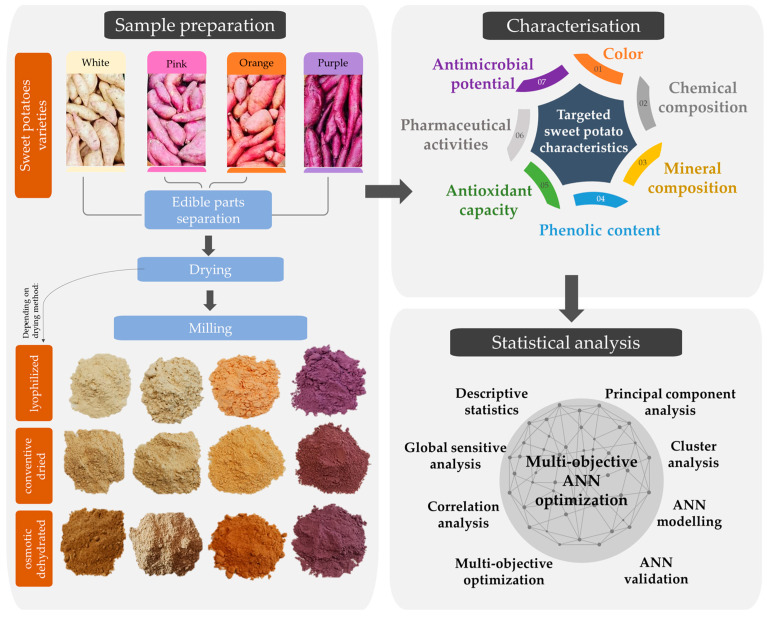
Graphical presentation of experimental setup.

To achieve the main goal of this study, simultaneous steps in the experimental work are established. Figure 1 presents a schematic review of three main steps: sample preparation, characterization, and statistical analysis.

### 2.1. Vegetable Material and Processing Steps

**Figure 2:**
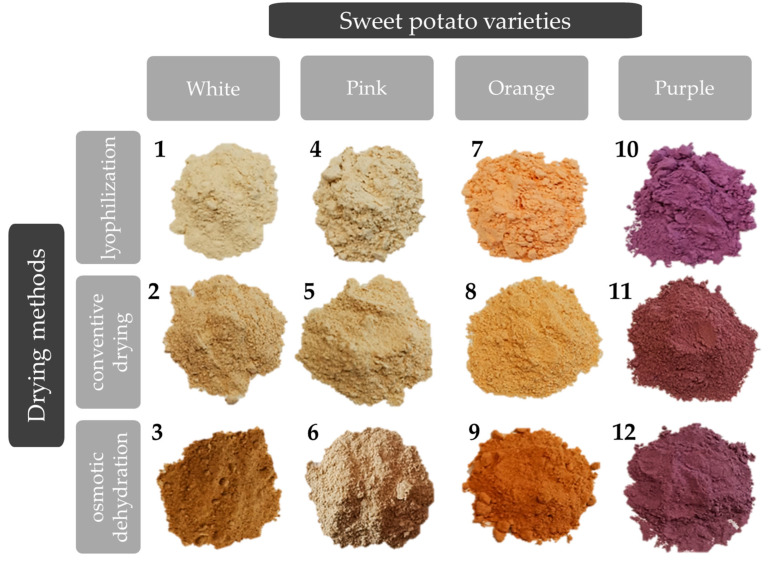
Dried samples of different sweet potatoes marked with numbers from 1 to 12 for easier presentation of the drying method-sweet potato variety combination.

The different sweet potatoes were obtained in 2022 from the family farm specialized in sweet potato production located in Kać, Novi Sad, Republic of Serbia (45.2993319° N, 19.9462154° E). For this study, four different sweet potato varieties were used, the main visual difference being in flesh color—white, pink, orange, and purple (Figure 1). Edible parts were separated (peeled) and dried using three different techniques: lyophilization, convective drying, and osmotic dehydration. The sweet potato samples were sliced into 0.5 cm thickness and 3.6 cm diameter with a Nemco slicer (accuracy of ±0.27 mm) (55200AN, Hicksville, OH, USA). The convective drying was performed in a single layer on a wire mesh tray, placed in a drying chamber at a temperature of 70 ± 1 °C until the constant weight. The osmotic pre-treatment was performed in an 80% solution of sugar beet molasses at room temperature for 5 h. After osmotic pre-treatment, samples were washed using distilled water, gently blotted with tissue paper, and left 48 h in the freezer at −40 °C to prepare them for the lyophilization step. The lyophilization process involved primary freezing of all samples at −40 °C for 2 h in the Martin Crist Alpha 2–4 (Osterode, Germany) freeze-drier, while the main drying process was performed at 0.01 bar at −40 to 20 °C for 48 h, and at the end the final drying lasted 4 h at 0.005 mbar at 20–30 °C. Then, all samples were milled using a laboratory girder and sieved using 72 then 71 mesh sieves for homogenization and preserved in the fridge until further use. The final forms of dried sweet potato samples are presented in Figure 2. This figure contains sample marks (numbers 1–12, depending on the drying method and sweet potato variety combination), which represent the data gained in the following steps.

### 2.2. Evaluation of Targeted Sweet Potato Characteristics

#### 2.2.1. Color of Sweet Potato

Sweet potato color was determined in triplicate using a Minolta Chroma Meter CR-400 colorimeter (Konica Minolta Sensing Inc., Tokyo, Japan) (diameter of contact surface 8 mm). Before the measurement, calibration was performed with a white color standard. The results are presented according to the CIELab color system, where the coordinates are defined as follows: L* is the color lightness coordinate (where 0 means black and 100 white), a* is the red-green coordinate (where a*+ means red and a*− indicates green color), b* is a yellow-blue coordinate (where b*+ indicates yellow and b*− indicates blue) [13].

#### 2.2.2. Chemical Composition

The sweet potato sample’ proximate composition, covering protein (Method No. 950.36), fat (Method No. 935.38), reducing sugar (Method No. 975.14), total dietary fiber (Method No. 958.29), ash (Method No. 930.22), and moisture content (Method No. 926.5), was evaluated using AOAC standard analysis methods (2000) [14].

#### 2.2.3. Mineral Composition

The potassium, sodium, calcium, magnesium, iron, copper, and zinc content were measured using an atomic absorption spectrophotometer (Varian SPECTRAA-10). The instrument’s parameters followed the manufacturer’s recommendations, utilizing thousand ppm standards from AccuStandard (New Haven, CT, USA) and calibration curves within a linear range (r = 0.999), according to the AOAC method [15].

The mineral composition of sweet potato was analyzed using AAS after a two-phase dry ashing process. Initially, 5 g of sweet potato sample was heated in a ceramic crucible on a hotplate until no fumes were observed. This crucible was transferred to a preheated muffle furnace at 550 °C for a 4-h ashing period, followed by cooling.

Subsequently, a 1:1 HNO_3_ mixture (5 mL) was added to the crucible and heated on the hotplate (120–150 °C) to evaporate the HNO_3_. The crucible was placed in the muffle furnace (550 °C) for another 4 h. If the resulting ash was not gray-whitish, adding HNO_3_ and heating was repeated. After the final cooling, the ash was dissolved: 1:1 HCl (10 mL) was cautiously added to the crucible and heated until 5 mL evaporated. The remaining 5 mL was transferred to a 50-mL flask with Cs, La, and water to the mark.

#### 2.2.4. Extracts Preparation

The first step to analyze the vast potential of four different color-fleshed sweet potatoes under various drying techniques was the extraction, performed according to Šeregelj et al. [16], with slight modifications. Namely, acetone: ethanol (36:64) mixture was used as a solvent, exposing plant materials to 1 min of vortex homogenization, followed by 20 min in an ultrasonic bath (RK 52 H, 1.8 L, SA GR, Lab Logistics Group GmbH, Meckenheim, Germany. After using a centrifuge (LACE-24, COLO Lab Experts Slovenia, Gramma Libero, Belgrade, Serbia) for 5 min at 4000 rpm, the supernatant was separated and stored until further use.

#### 2.2.5. Total Phenolic Content

For the determination of total phenolic content, the microscale-adapted -Folin-Ciocalteau method was used for all extracts, as described in the research of Šovljanski et al. [17]. In particular, 170 μL of distilled water, 15 μL of extracts/or blank (water), 12 μL of -Folin-Ciocalteau reagents, and 30 μL of sodium carbonate were mixed in a microtiter plate and left in the dark for 1 h. Results were obtained using spectrophotometry on a wavelength of 750 nm and expressed as gallic acid equivalents (GAE) per 100 g of dried sample.

#### 2.2.6. Antioxidant Capacity

The antioxidant potential of different sweet potato samples was investigated spectrophotometrically using four assays: 2,2-diphenyl-1-picrylhydrazyl (DPPH^●^), 2,2′-azino-bis-3-ethyl benzo-thiazoline-6-sulphonic acid (ABTS^●+^), reducing power (RP), and superoxide anion method (SoA), on different wavelengths (515 nm, 414 nm, 750 nm, and 540 nm, respectively).

In short, the protocol for the DPPH assay was followed by Aborus et al. [18]. A microplate well was filled with 250 μL of the DPPH^•^ methanol solution and 10 μL of diluted samples and placed in a dark area for 50 min, using methanol as a blank before measurement. For the ABTS^●+^ test, absorbances of ABTS^●+^ solution (250 μL) were measured before and after 35 min of addition of 3 mL of investigated extracts as described by Šeregelj et al. [19]. Water was used as blank, and absorbances were calculated using Equation (1).

(1) $$\Delta A=A_{0}-A_{fin}-A_{blank}$$

Using a 96-well microplate adaptation of Oyaizu’s approach [20], reducing power (RP) was ascertained. To sum up, 25 μL of potassium iron (III) cyanide (1%), 25 μL sodium phosphate buffer (pH = 6.6), and 25 μL water were combined for the blank test. Following a 20-min incubation period at 50 °C in a water bath, 25 microliters of 10% trichloroacetic acid were added once it had cooled down. From this solution, 50 μL was taken out and added to the microplate along with 50 μL of distilled water and 10 μL of 0.1% iron (III) chloride.

The SoA method was performed as in the work of Girones-Vilapana [21]. It included monitoring the effects of controls and samples on the O_2_^•−^ radical induced by reduction in NBT. The NADH/PMS system generated superoxide radicals. All reagents were dissolved in the 7.4 pH phosphate buffer, including NADH (β-nicotinamide adenine nucleotide reduced disodium salt hydrate), NBT (nitro blue tetrazolium), and PMS (phenazine methosulfate). The experiment in triplicate was performed, expressing. Compared to the reduction in NBT, percentage control was calculated before being and expressed as Trolox equivalent (Equation (2)).

(2) $$\%\mathrm{Inhibition}=\frac{\left(\Delta A_{control}-\Delta A_{sample}\right)}{\Delta A_{control}}\cdot 100$$

Calibration curves were constructed for four antioxidant tests using Trolox. For different assays from different wavelengths, results were obtained as mM Trolox equivalents per 1 mL and calculated as mM Trolox equivalents in 100 g of dried weight (DW).

#### 2.2.7. Pharmaceutical Activities

For pharmaceutical activities of different sweet potato extracts, anti-inflammatory (AIA) and anti-hyperglycemic (AHgA) activities were utilized as in detail explained in the study of Ranitović et al. [22]. Percentages of inhibitory activities were calculated using absorbances obtained from a microtiter plate reader (Thermo Fisher Scientific Inc., Waltham, MA, USA) of samples and their controls at the concentration of 50 mg/mL for all extracts. More specifically, for AIA, a mixture of egg albumin (0.2 mL), 6.4 pH phosphate puffer (2.8 mL), and extract (2 mL) was incubated at two different temperatures (37 °C and 70 °C) for a total of 20 min before measuring protein denaturation. Sample and control (water) absorbances were measured at a wavelength of 660 nm and calculated according to Equation (3).

(3) $$\mathrm{AIA}(\%)=\frac{\left(A_{control}-A_{sample}\right)}{A_{control}}\cdot 100$$

As for AHgA, after diluting substrate (4-nitrophenyl α-D-glucopyranoside) and enzyme (α-glucosidase) in 10 mM potassium phosphate buffer pH 7.2, preparation of reaction mixtures involved adding 100 μL of substrate solution, 20 μL of sample, and 100 μL of enzyme solution. A control blank and sample blank, without added enzyme, were also prepared, and everything was measured before and after 10 min of incubation (37 °C) at 405 nm (Equation (4)).

(4) $$\mathrm{AHgA}(\%)=\frac{\left(\Delta A_{control}-\Delta A_{sample}\right)}{\Delta A_{control}}\cdot 100$$

#### 2.2.8. Antimicrobial Potential

To ascertain the antimicrobial profile of the extracts, disc diffusion tests were conducted utilizing the following bacterial strains: Gram-negative bacteria (*Escherichia coli* ATCC 25922 and *Pseudomonas aeruginosa* ATCC 27853) and Gram-positive bacteria (*Staphylococcus aureus* ATCC 25923 and *Enterococcus faecalis* ATCC 19433). *Candida albicans* ATCC 10231 and *Aspergillus brasiliensis* ATCC 16404 were chosen as representatives of yeast and fungi, respectively. -Mueller-Hinton or Sobouraud maltose agar was inoculated with microbial suspensions containing approximately 6 log CFU/mL [23]. Subsequently, 15 μL of the extract, at a concentration of 50 mg/mL, was added to three sterile discs with a diameter of 6 mm. Sterile distilled water was employed as the negative control, while clavulanic acid (Sigma-Aldrich, St. Louis, MI, USA) and cycloheximide (Acros Organic, New Jersey, NJ, USA) were utilized as the positive controls. Interpretation of the results was based on the size of the inhibition zones, classified as sensitive (if the zone diameter exceeded 26 mm), intermediate (for diameters ranging from 22 to 26 mm), and resistant (if the diameter was less than 22 mm).

### 2.3. Statistical Analysis

The experimental data underwent statistical analysis utilizing a variety of multivariate mathematical techniques, including descriptive statistics, principal component analysis (PCA), cluster analysis, Artificial Neural Network modeling, and global sensitivity analysis. These analyses were conducted using StatSoft Statistica 10.0^®^ software. Additionally, a color plot diagram was created using R software version 4.0.3 (64-bit), employing the “circle” method with an upper-type configuration.

#### 2.3.1. ANN Modeling

An artificial neural network model (ANN), specifically a multi-layer perceptron (MLP) with three layers (input, hidden, and output), was employed to predict total phenolic contents (Phenols), antioxidant capabilities through DPPH, ABTS, RP, SoA assays, pharmacological activity through AIA and AHgA, color properties through a*, b*, and L* values, chemical composition (moisture, sugars, fats, proteins, cellulose, total carbs, and ash), and mineral composition (Na, Fe, K, Ca, and Mg content) based on the variety of sweet potato and drying method. The existing literature has demonstrated the effectiveness of ANN models in approximating nonlinear functions [24,25,26]. Before commencing the calculations, input and output data were normalized to enhance the ANN’s performance. In an iterative process, the input data were repeatedly presented to the network [27]. The Broyden–Fletcher–Goldfarb–Shanno (BFGS) algorithm was the iterative method for solving unconstrained nonlinear optimization within the ANN modeling.

**Figure 3:**
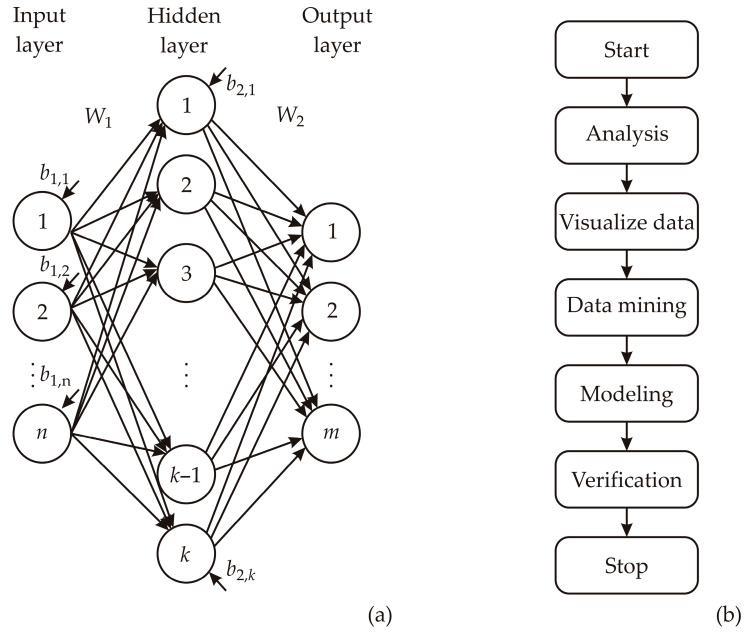
ANN model building: (**a**) ANN scheme, (**b**) flowchart of the conducted research.

Figure 3 schematically illustrates the ANN model structure and the ANN building flowchart aimed at identifying the optimal ANN model in terms of both predictive capability and error rates associated with each model.

Coefficients linked to the hidden layer, encompassing both weights and biases, were organized into matrices denoted as *W*_1_ and *B*_1_. Correspondingly, coefficients associated with the output layer were grouped in matrices designated as *W*_2_ and *B*_2_. This neural network can be represented using matrix notation, where *Y* represents the matrix of output variables, *f*_1_ and *f*_2_ denote the transfer functions in the hidden and output layers, respectively, and *X* stands for the matrix of input variables [28]

(5) $$Y=f_{1}(W_{2}\cdot f_{2}(W_{1}\cdot X+B_{1})+B_{2})$$

The weight coefficients, which are the elements of matrices *W*_1_ and *W*_2_, were determined throughout the ANN learning process. Optimization procedures were applied to update these coefficients, minimizing the error between the network’s predictions and experimental outputs. This optimization process involved the sum of squares (SOS) and utilized the Broyden–Fletcher–Goldfarb–Shanno (BFGS) algorithm to expedite and stabilize convergence, as established by Suszyński and Peta [29] and Stojić et al. [30]. Coefficients of determination served as parameters to assess the performance of the resulting ANN model.

#### 2.3.2. Global Sensitivity Analysis

Yoon’s global sensitivity equation was employed to assess the influence of input parameters on output variables based on the weight coefficients derived from the developed ANN [31]:

(6) $$RI_{ij}(\%)=\frac{\sum_{k=0}^{n}\left(w_{ik}\cdot w_{kj}\right)}{\sum_{i=0}^{m}\left|\sum_{k=0}^{n}\left(w_{ik}\cdot w_{kj}\right)\right|}\cdot 100\%$$

where *w*—weight coefficient in the ANN model, *i*—input variable, *j*—output variable, *k*—hidden neuron, *n*—number of hidden neurons, and *m*—number of inputs.

#### 2.3.3. Multi-Objective Optimization

The developed artificial neural network (ANN) model investigated for a crucial role in a multi-objective optimization (MOO) scenario. Its purpose was to identify the optimal sweet potato variety (white, orange, pink, and purple) and drying method (convective, osmotic, and lyophilization) that minimizes alterations in TPC, DPPH, ABTS, RP, SoA, anti-inflammatory activity, and anti-hyperglycemic activity. To tackle this multi-objective optimization problem, a Pareto front was established using a specific objective function, as explained by Kojić et al. [32]. The solution space of the MOO was explored using a genetic algorithm (GA) based on the approach described by Goldberg in 1989 [32]. In the GA, populations were evaluated using a distance measure for each point in the current generation, a technique detailed by [23,32,33,34].

#### 2.3.4. The Accuracy of the Model

The numerical validation of the generated model was tested employing the coefficient of determination (*r*^2^), reduced chi-square (*χ*^2^), mean bias error (*MBE*), root-mean-square error (*RMSE*), and mean percentage error (*MPE*), calculated as follows [35]:

(7) $$\chi^{2}=\frac{\sum_{i=1}^{N}{\left(x_{\exp,i}-x_{pre,i}\right)}^{2}}{N-n}$$

(8) $$RMSE={\left[\frac{1}{N}\cdot \sum_{i=1}^{N}{\left(x_{pre,i}-x_{\exp,i}\right)}^{2}\right]}^{1/2}$$

(9) $$MBE=\frac{1}{N}\cdot \sum_{i=1}^{N}\left(x_{pre,i}-x_{\exp,i}\right)$$

(10) $$MPE=\frac{100}{N}\cdot \sum_{i=1}^{N}\left(\frac{\left|x_{pre,i}-x_{\exp,i}\right|}{x_{\exp,i}}\right)$$

where *x_exp,__i_* stands for the experimental values and *x_pre,__i_* are the predicted values calculated using the model, *N* and *n* are the number of observations and constants, respectively.

## 3. Results and Discussion

The primary objective of this research article is to explore the optimization potential of artificial neural networks (ANNs) in the context of the influence of drying methods of different sweet potato varieties and impacts on targeted characteristics: color, chemical composition, mineral composition, phenolic content, antioxidant capacity, pharmaceutical activities as well as antimicrobial potential.

### 3.1. Color Characteristics of Sweet Potato Samples

Since the color of sweet potatoes has been proven to play a major role in their beneficial effect on health [4], Table 1 represents the results obtained from the color properties of four sweet potato varieties. The color measurements are assessed using the CIE Lab color space coordinates, encompassing L* (lightness), a* (red-green), and b* (yellow-blue). Three drying methods, lyophilization, convective drying, and osmotic dehydration, are employed to examine potential variations in the color profiles of white, pink, orange, and purple sweet potato varieties. The mentioned drying method significantly influences the color characteristics of various sweet potato varieties. In the case of white sweet potato, lyophilization yields the highest L* value, indicating increased lightness, while osmotic dehydration results in the lowest L* value, suggesting diminished lightness. The transition of a* values from negative (lyophilization) to positive (convective drying and osmotic dehydration) signifies a shift from green to red tones. Concurrently, b* values increase from lyophilization to osmotic dehydration, implying a progression towards more yellow tones.

For pink samples, lyophilization and convective drying yield similar L* values, while osmotic dehydration leads to a lower L* value, indicating reduced lightness. The a* values increase from lyophilization to osmotic dehydration, suggesting a transition towards red tones. Additionally, b* values increase from lyophilization to osmotic dehydration, signifying a shift towards more yellow tones. The orange variety exhibits the highest L* value with lyophilization and the lowest with osmotic dehydration. The a* values decrease from lyophilization to osmotic dehydration, indicating a shift from red to green tones, while b* values increase from lyophilization to osmotic dehydration, suggesting a progression towards more yellow tones. In the case of purple sweet potato samples, lyophilization and convective drying result in similar L* values, while osmotic dehydration leads to a slightly lower L* value. The a* values increase from lyophilization to osmotic dehydration, signifying a shift towards red tones, whereas b* values decrease from lyophilization to osmotic dehydration, indicating a transition towards more blue tones.

So far, there are still no studies focused on the assessment of different drying methods of various sweet potato species. In the work of Hariadi et al. [36], lyophilization was picked out of three investigated drying methods of purple sweet potato from Indonesia for its best characteristics, including color intensity L* 64.20, a*, 65.60, and b* 18.52. Although the L* value is in accordance with the result of this study for the lyophilized purple sample, the other two color parameters are significantly different. Furthermore, the osmotic dehydration process was used on orange-fleshed sweet potato in China with the following color parameters: L* 64.79, a* 32.71, b* 42.8 [37], showing similar L* values with the present study, but vastly different b* value. A researcher from Turkey [38] optimized the convective drying parameters of sweet potato, obtaining the highest L* (73.3) values measured for the sample dried at 60 °C. L* value in this research varied from 55.85 to 85.75, showing a similar range. It can be summarized that the drying method significantly influences the color properties of sweet potatoes, with distinct patterns observed for each type. The variations in the color characteristics of sweet potatoes provide insights into the impact of drying methods, potentially directing optimization strategies for preserving or enhancing specific color attributes in the drying process for utilization in specific food industry needs.

### 3.2. Chemical Analysis of Sweet Potato Samples

The next step involved investigation of the chemical composition of sweet potato samples (Table 2). The chemical composition parameters included moisture content, proteins, fats, total sugars, cellulose, ash, and total carbohydrates, expressed as percentages of dry weight (% DW). In the case of white sweet potato samples, lyophilization resulted in a moisture content of 5.12%, while convective drying and osmotic dehydration yielded higher moisture levels of 6.03% and 7.1%, respectively. The protein content remained relatively consistent across drying methods, with convective drying exhibiting the highest protein content at 13.07% and lyophilization the lowest at 12.32%. The fat content varied, with osmotic dehydration recording the lowest value at 0.55%. Total sugars, cellulose, ash, and total carbohydrates exhibited fluctuations based on the applied drying method. Similarly, lyophilization for the pink variety resulted in a lower moisture content (3.2%) compared to convective drying (4.61%) and osmotic dehydration (9.05%). Convective drying recorded the highest protein content (14.27%), while osmotic dehydration resulted in the lowest (13.4%). Fats, total sugars, cellulose, ash, and total carbohydrates demonstrated varying trends across drying methods (Table 2). In the case of orange samples, lyophilization yielded the lowest moisture content (3.48%), whereas osmotic dehydration led to the highest (7.93%). Convective drying resulted in the highest protein content (12.04%), while osmotic dehydration recorded the lowest (10.5%). Fluctuations are also observed in fats, total sugars, cellulose, ash, and total carbohydrates. In the case of purple samples, lyophilization and convective drying showed similar trends in moisture content, protein, fats, total sugars, cellulose, ash, and total carbohydrates. On the other hand, osmotic dehydration of purple sweet potato introduced substantial variations, particularly evident in moisture content (5.53%) and total carbohydrates compared with other drying methods.

Rodrigues et al. [39] determined the chemical composition of orange and purple-fleshed sweet potatoes and their flour, obtained by hot air drying at 65 °C. Respectively, results of dried orange and purple sweet potato samples included: moisture (10.97, 6.91%), ash (22.11, 3.07%), protein (4.90, 5.82%), fats (0.39, 0.39%), and total carbohydrates (90.13, 88.15%). In comparison, the drying methods used in this research obtained lower moisture levels (except osmotic dehydration on the purple sample) and lower ashes content, while higher levels of protein, fats, and carbohydrates were exhibited. The observed variations in the chemical composition parameters underscore the distinct effects of drying methods on the nutritional components of sweet potato varieties, providing valuable insights for both food processing and nutritional considerations.

### 3.3. Minerals Content of Sweet Potato Samples

As shown in Table 3, the mineral composition, including potassium (K), magnesium (Mg), calcium (Ca), iron (Fe), and sodium (Na) was defined. The lyophilized white sweet potato resulted in elevated potassium levels (26,140.70 mg/kg) compared to convective drying and osmotic dehydration. Convective drying demonstrated the highest magnesium (1358.60 mg/kg) and iron content (33.41 mg/kg), while osmotic dehydration enabled the highest calcium (2665.09 mg/kg) and sodium content (14,272.88 mg/kg). In the case of the pink variety, higher potassium levels (27,530.03 mg/kg) were obtained for lyophilization than other drying methods, while osmotic dehydration led to the highest sodium content (13,368.26 mg/kg). Convective drying resulted in the highest magnesium (1116.89 mg/kg), calcium (1975.16 mg/kg), and iron (24.61 mg/kg) levels. For orange samples, the highest potassium levels (28,030.08 mg/kg) were observed during lyophilization, while osmotic dehydration produced the highest sodium content (11,070.10 mg/kg). Convective drying enabled elevated levels of magnesium (899.41 mg/kg), calcium (1840.84 mg/kg), and iron (35.06 mg/kg). In the case of the purple variety, lyophilization possessed higher potassium levels (19,427.35 mg/kg), while osmotic dehydration led to the highest sodium content (6919.21 mg/kg). Convective drying demonstrated higher levels of magnesium (1025.13 mg/kg) and calcium (1786.42 mg/kg), while lyophilization resulted in the highest iron content (21.86 mg/kg). The notable differences in mineral composition between samples were obtained using osmotic dehydration as pre-treatment.

Osmotic dehydration involves the removal of water from a food product by immersing it in a hypertonic solution, typically a sugar or salt solution. The leaching effect in osmotic dehydration refers to the loss of soluble components such as sugars, minerals, vitamins, and flavor compounds from the food product into the osmotic solution. While the primary goal of osmotic dehydration is to remove water from the food, this process can also cause the migration of these soluble components [8].

However, sugar beet molasses is a complex osmotic medium with a rich nutritional composition, causing soluble compounds to enter the sample during the osmotic dehydration process and affecting its chemical composition. Permeability of the food matrix can affect the rate and extent of change in the chemical composition, causing different results for different types of sweet potatoes.

As an essential mineral nutrient, K content was measured in yellow sweet potato after the convective drying process, presenting significantly lower results than in this study, with 788 and 725 mg/100 g at temperatures of 50 °C and 70 °C, respectively [40]. Also, in the research of Grabowski et al. [41], sweet potato puree was spray-dried in the form of powder, with control of dried samples with no pre-treatments showing mineral content of 151 mg/100 g Ca, 99 mg/100 g Mg, 1822 mg/100 g K, 4.8 mg/100 g Fe, and 122 mg/100 g Na, showing comparable results. The results highlighted the significant influence of both sweet potato variety and drying method on the mineral composition, which strongly contributes to a comprehensive understanding of the nutritional characteristics of sweet potatoes based on varied drying techniques.

### 3.4. Bioactive Compounds and Biological Potentials of Sweet Potato Samples

As shown in Table 4, depending on the variety of sweet potato, more so than the drying method, the total phenolics’ presence ranges from 191.44 to 2006.91 mg/100 g. For the white sweet potato variety, the lyophilization method yielded the lowest total phenolic content (191.44 ± 9.80 mg/100 g DW), while convective drying and osmotic dehydration resulted in higher values (278.24 ± 16.17 and 283.06 ± 17.35 mg/100 g DW, respectively). Similarly, in the case of pink sweet potatoes, lyophilization yielded a lower content (182.75 ± 10.12 mg/100 g DW) compared to convective drying (207.45 ± 5.63 mg/100 g DW) and osmotic dehydration (255.29 ± 7.16 mg/100 g DW). The orange sweet potato variety exhibited a distinctive trend, with lyophilization resulting in the highest total phenolic content (376.64 ± 27.89 mg/100 g DW), while convective drying and osmotic dehydration showed lower values (211.55 ± 11.78 and 227.53 ± 17.13 mg/100 g DW, respectively). Notably, the purple sweet potato variety consistently displayed the highest total phenolic content across all drying methods. Lyophilization, convective drying, and osmotic dehydration resulted in substantial values of 1677.76 ± 61.23, 1428.23 ± 82.94, and 2006.81 ± 113.7 mg/100 g DW, respectively. This emphasizes the potent phenolic profile of purple sweet potatoes, making them a robust source of these beneficial compounds. The observed variations in total phenolic content among different sweet potato varieties and drying methods provide valuable insights into the potential impact of these factors on the nutritional composition of *I. batatas.* Some similarities and differences in the influence of sweet potatoes and drying methods can be defined by comparing the literature. Purple-fleshed sweet potato is by far the strongest carrier of phenolic compounds among other color varieties, confirmed by several studies. In the research of Chintha et al. [42], purple-fleshed sweet potato was found to possess the highest total phenolic content with 2.1 and 2.84 mg GAE/100 g FW found, respectively, in ethanol and aqueous extracts. Namely, total phenols in the amount of 179.8 mg GA/100 g in a dried orange sample obtained using the same solvent mixture was obtained by Šeregelj et al. [16], while similar research [43] indicated 266.39 mg GA/100 g of total phenolics in an ethanol solution of *I. batatas*.

The antioxidant potential is highly related to the total phenolic content. Following the same trend as total phenolic compounds, in all four antioxidant assays, the strength of the samples’ scavenger activity can be observed in descending order: purple > orange > pink > white-skinned *I. batatas* sample. Optimal drying methods for retaining the highest antioxidant potential would be complicated to determine because of the different effects of drying methods on different sweet potato varieties, as well as for various antioxidant tests. As can be seen in Table 4, a wide range of values for antioxidant activity was obtained, depending on the drying method used, flesh color, and antioxidant assay can be observed. Purple samples consistently exhibit the highest antioxidant activity across all assays and drying techniques, showcasing their potential as a rich source of antioxidants. For white sweet potatoes, convective drying resulted in the highest DPPH^●^ and SoA values, suggesting increased free radical scavenging capacity. Osmotic dehydration, on the other hand, demonstrated notable ABTS^●+^ and RP activities, highlighting its effectiveness in certain antioxidant assays. Pink sweet potatoes exhibited consistent antioxidant activity across different drying methods. Convective drying resulted in the highest ABTS^●+^ and SoA values, indicating strong free radical scavenging potential. Osmotic dehydration showed notable DPPH^●^ and RP activities, suggesting its effectiveness in specific assays. Orange sweet potatoes displayed distinctive antioxidant activity patterns. Lyophilization demonstrated the highest ABTS^●+^ and SoA values, indicating potent free radical scavenging ability. Convective drying showed substantial ABTS^●+^ and RP activities, while osmotic dehydration exhibited notable DPPH^●^ and RP activities. Numerous researchers investigated the antioxidant activity of *I. batatas* tubers, including Ji et al. [44], where 43.3–81.2 Mm TE/g of scavenger activity against DPPH radicals was reported for four different colored *I. batatas*. Also, hot air drying of sweet potato samples while varying temperatures from 60 to 110 °C [45] was performed to determine the optimal drying conditions for the preservation of present antioxidants, obtaining the highest result of ORAC antioxidant activity of 130.94 μmol TE/g during the temperature of 80 °C.

Varieties of sweet potatoes with brightly colored skin are especially interesting because of the presence of their natural pigments and their connection to antidiabetic, hepatoprotective, antioxidative, and anti-cancer activity, and even more, their use in the food industry as natural pigments, antioxidants, and supplements [46]. Therefore, Table 4 also summarizes the gained results for two pharmacological potentials: anti-inflammatory activity (AIA) and anti-hyperglycemic activity (AHgA). To determine these pharmacological potentials of various *I. batatas*, inhibition of α-amylase and protein denaturation was measured, and the results indicate lower to moderate activities. Purple sweet potato has the lowest AIA and AHgA values, while orange and pink-skinned batatas have the highest AIA (58.72 and 56.81%). Furthermore, white-skinned sweet potato has the strongest AHgA (61.56%). Based on the data presented in Table 4, the convective drying technique seems to be the method of choice for maintaining and determining the pharmaceutical activities of sweet potato samples. Depending on the type of extracts and batatas cultivators, α-amylase enzyme inhibitory activity varied from 56.85 to 98.78% in the research of Chintha et al. [43]. AHgA natural property of sweet potatoes is considered to be based on their phytochemical content, correlated to the presence of flavones. According to Ayeleso et al. [3], multiple reports associate white-skinned *I. batatas* with high anti-hyperglycemic activity, which agrees with this research’s results. Also, many studies demonstrated the correlation between the inhibition of lipopolysaccharide-inducing inflammatory responses and the intake of sweet potatoes rich in biomolecules [3].

Furthermore, antimicrobial testing of four varieties of sweet-fleshed potatoes was done following the appearance of inhibition zones during contact between tested sweet potato samples and microbial strains. All investigated samples exhibited no growth-suppressing effect (inhibition zones were not detected) on the selected fungi, yeasts, as well as both Gram-positive and Gram-negative bacteria (*A. brasiliensis*, *C. albicans*, *S. aureus*, *E. faecalis*, *E. coli*, and *P. aeruginosa*). While there is a lack of reports on the antimicrobial properties of sweet potato flesh, previous research has supported microbial evaluation of sweet potato leaves and roots. Lee et al. [47] assessed the antimicrobial activity of a water extract derived from spray-dried sweet potato roots against three Gram-positive bacteria (*Staphylococcus epidermidis*, *Staphylococcus aureus*, *Enterococcus faecalis*) and two Gram-negative bacteria (*Bacillus cereus*, *Escherichia coli*), noting that *Staphylococcus* species exhibited the greatest zones of growth inhibition. Since this study did not involve information about sweet potato variety, a deeper correlation with the results gained in this study is not considered. Furthermore, Naz et al. [48] found that orange sweet potato varieties, extracted using methanol and ethanol, exhibited antimicrobial activity against Gram-positive *P. multocida* bacteria, while none of the tested extracts inhibited the growth of *E. coli*. The absence of inhibition zones observed in the case of *S. aureus*, *E. faecalis*, *E. coli*, *C. albicans*, and *Aspergillus brasiliensis* in this study can likely be attributed to the non-specific selection of solvents, resulting in the insufficient isolation of chemical compounds capable of inhibiting the tested microorganisms. Considering that the tested samples showed no antimicrobial potential, these results were omitted from the following statistical analysis.

### 3.5. Color Correlation of the Sweet Potato Samples

**Figure 4:**
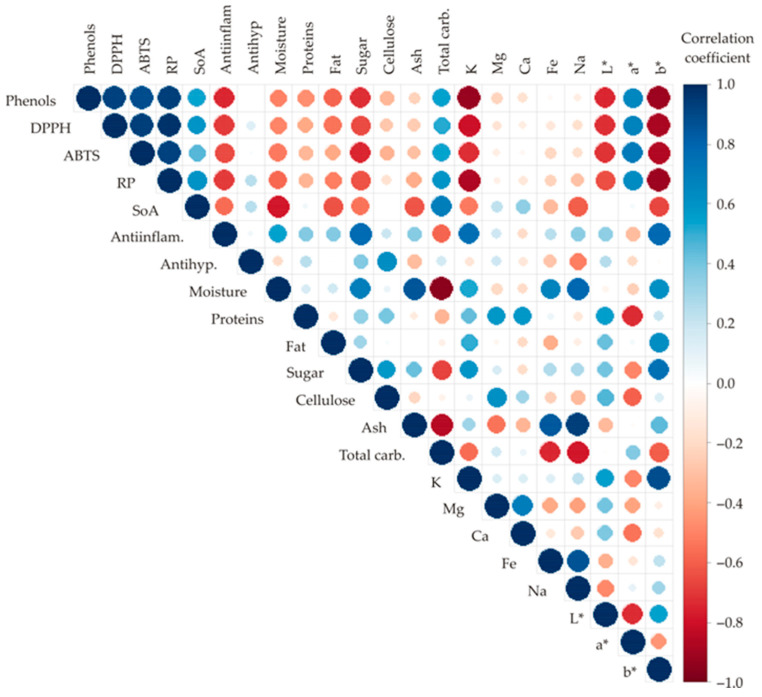
Color correlation graph between observed responses.

Figure 4 presented a color correlation graph between all tested outputs for sweet potato characterization. Statistically significant correlations (*p* ≤ 0.05) were found between most of the analyzed responses. The size and the circle’s color depend on the correlation coefficients; if the color is blue, the positive correlation is achieved, and on the contrary, the red color represents the negative correlation. Also, the circle‘s size is increased with the absolute value of the correlation coefficient.

The highest positive correlations were found between phenolics and RP (r = 0.939), phenolics and DPPH (r = 0.927), and phenolics and ABTS (r = 0.882). Furthermore, DPPH was positively correlated to ABTS (r = 0.938) and RP (r = 0.971), while RP was also positively correlated with ABTS (r = 0.923). On the other hand, the highest negative correlations were observed between the content of carbs and moisture (r = −0.957) and phenolics and K (r = −0.925).

### 3.6. Principal Component Analysis

**Figure 5:**
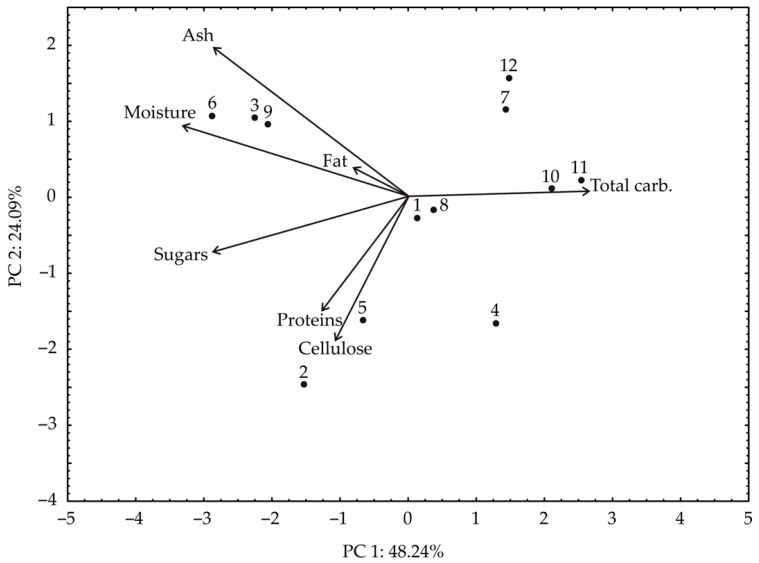
The PCA biplot diagram of the relationships among moisture, sugars, proteins, cellulose, total carbs, fats, and ash content. The samples are numbered according to Table 1.

The PCA biplot of the relationships among ash, moisture, fats, sugars, proteins, cellulose, and total carbon-hydrates content revealed that the first two principal components explained 72.33% of the total variance in the observed parameters (Figure 5). PCA analysis has shown a good grouping of sweet potato samples according to the drying method. According to the results of the PCA, the content of total carbon-hydrates (which contributed to 27.7% of the total variance, based on correlations) showed a positive influence on the PC1 coordinate. On the other hand, the content of moisture (27.3%), sugars (20.2%), and ash (19.1%) negatively influenced the calculation of the PC1. The positive influence on the PC2 coordinate was noticed for ash content (17.3% of the total variance, based on correlations), while the negative influence on the PC2 coordinate was determined for proteins (26.3%), sugars (9.6%), and cellulose content (43.5%).

The lyophilized white, pink, and orange sweet potato samples (1, 4, and 7), the convective dried white, pink, and orange sweet potato samples (2, 5, and 8), osmotic dried white, pink, and orange sweet potato samples (3, 6, and 9) were grouped in the factor space according to their similarities in chemical composition. The purple sweet potato samples (10, 11, and 12) were grouped regardless of the drying method.

**Figure 6:**
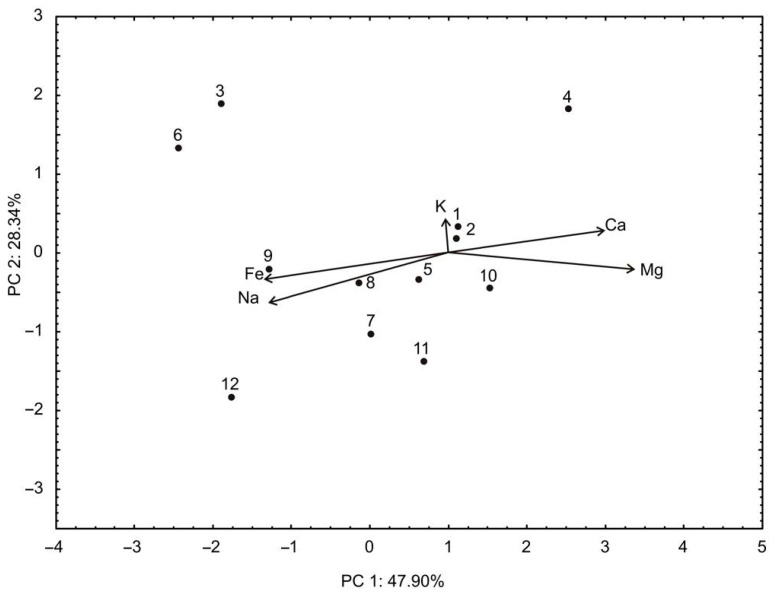
The PCA biplot diagram of the relationships between mineral content. The samples are numbered according to Table 1.

According to the PCA analysis of the relationships among K, Mg, Ca, Fe, and Na content (Figure 6), the first two principal components explained 76.24% of the total variance in the observed parameters. According to the results of the PCA, the content of Ca (which contributed 15.8% of the total variance, based on correlations) and Mg showed a positive influence on the PC1 coordinate. On the other hand, the content of Fe (27.5%) and Na (31.1%) negatively influenced the calculation of the PC1. The positive influence on the PC2 coordinate was noticed for K content (33.7% of the total variance, based on correlations) and Ca (26.9), while the negative influence on the PC2 coordinate was determined for Mg (15.5%), Fe (12.7%), and Na content (11.2%). Moreover, the results of cluster analysis performed for the content of Na, Fe, K, Ca, and Mg are shown in Figure 6.

**Figure 7:**
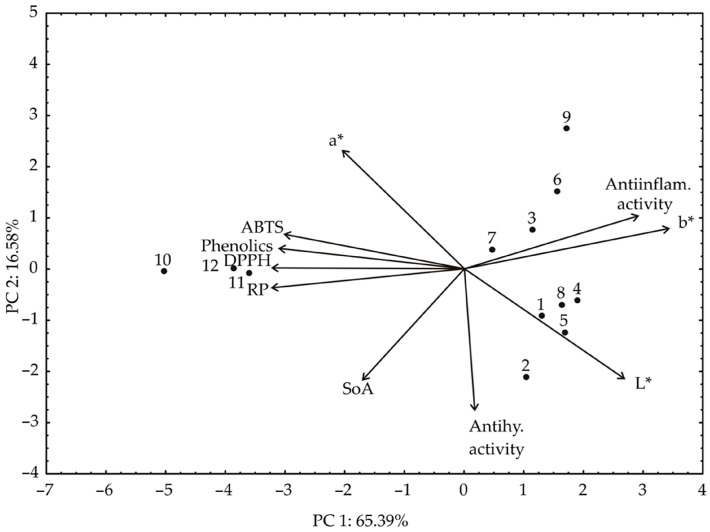
The PCA biplot diagram of the relationships among phenolics, DPPH, ABTS, RP, SoA, anti-inflammatory activity, anti-hyperglycemic activity, a*, b*, and L* values. The samples are numbered according to Table 1.

Based on the experimental results or color properties (Table 1) and biological compounds content and biological activities (Table 4), PCA analysis was performed (Figure 7).

Distinctive color variations of sweet potatoes arise from their unique nutrient profile, connecting their color with different bioactive properties. The yellow and cream-fleshed *I. batatas* varieties contain phenols and β-carotenes, while the red-fleshed variety has anthocyanins. However, the quantity of these nutrients is incomparable to the orange and purple-fleshed yams that accumulate high levels of carotenoids and anthocyanins, respectively. These nutrients play a crucial role in their antioxidant potential [49]. The PCA biplot of the relationships among phenolics, DPPH, ABTS, RP, SoA, AIA, AHgA, a*, b*, and L* values revealed that the first two principal components explained 81.97% of the total variance in the observed parameters. According to the results of the PCA, the content of phenolics, DPPH, ABTS, RP, Anti-inflammatory activity (%), and b* (which contributed 14.48%, 14.69%, 13.78%, 14.45%, 8.90%, and 13.28% of the total variance, based on correlations, respectively) showed a positive influence on the PC1 coordinate. On the other hand, the content of SoA (26.46%), anti-hyperglycemic activity (%) (28.61%), L*(19.92%), and a* (18.38%) positively influenced the calculation of PC2.

### 3.7. Cluster Analysis of Sweet Potato Samples

**Figure 8:**
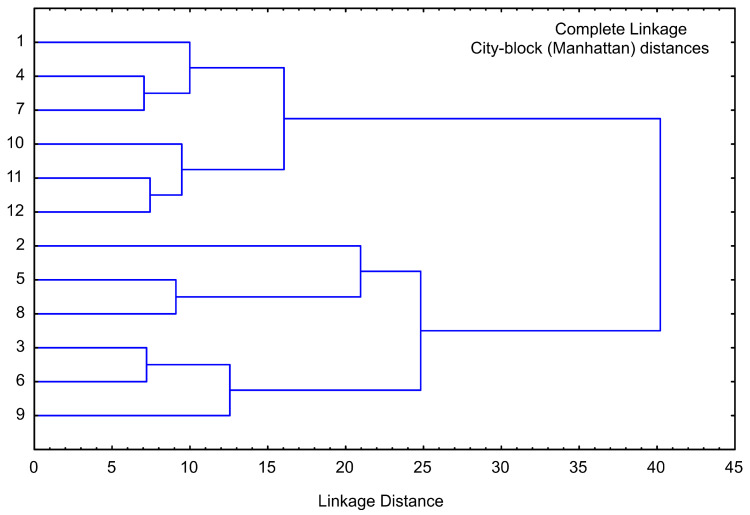
Cluster analysis of moisture, sugars, proteins, cellulose, total carbs, and ash. The samples are numbered according to Table 1.

Cluster analysis is performed for the same chemical composition parameters as for PCA analysis, and the obtained graph is given in Figure 8. The cluster analysis of chemical analysis showed four main clusters: the first cluster contained samples 1, 4, and 7 (lyophilized white, pink, and orange sweet potato samples), and the second cluster included samples 10, 11, and 12 (purple sweet potato samples), the third cluster contained samples 2, 5, and 8 (convective dried white, pink, and orange sweet potato samples), and finally the fourth cluster included samples 3, 6, and 9 (osmotic dried white, pink, and orange sweet potato samples). The linkage distance between the main clusters was nearly 40.

**Figure 9:**
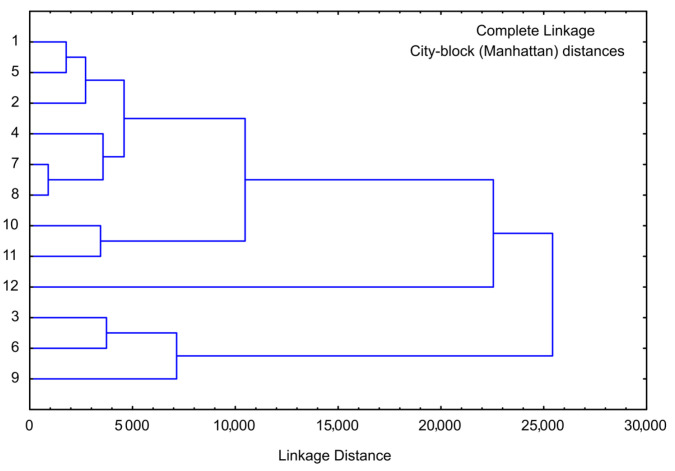
Cluster analysis of the content of Na, Fe, K, Ca, and Mg for the sweet potato samples. The samples are numbered according to Table 1.

Cluster analysis was also conducted for minerals data (employed in PCA analysis), Figure 9. PCA identified four primary clusters. The first cluster comprised samples 1, 2, and 5, while the second cluster encompassed samples 4, 7, and 8. Those two clusters cover white, pink, and orange sweet potato samples that were lyophilized or convective dried. Samples 10, 11, and 12 constituted the third cluster (purple sweet potato samples, regardless of the drying method), and the fourth cluster included samples 3, 6, and 9 (osmotic dried white, pink, and orange sweet potato samples). The linkage distance between these main clusters was approximately 25,000.

**Figure 10:**
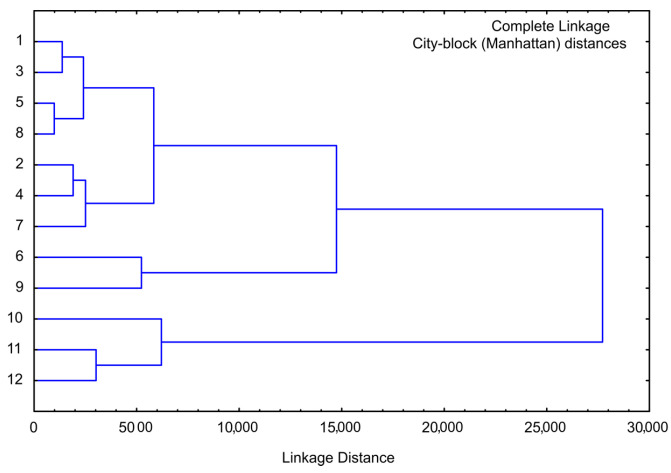
Cluster analysis of the observed samples in the context of the phenolics (mg GA/100 g), DPPH, ABTS, RP, SoA, anti-inflammatory activity (%), anti-hyperglycemic activity (%), a*, b*, and L* values. The samples are numbered according to Table 1.

The results of cluster analysis performed for the Phenolics (mg GA/100 g), DPPH, ABTS, RP, SoA, AIA activity (%), AHgA (%), a*, b* and L* values for the observed samples are given in Figure 10. The cluster analysis dendrogram revealed four separate clusters: the first cluster contained samples 1, 3, 5, and 8; the second cluster included samples 2, 4, and 7; the third cluster contained only samples 6 and 9; and finally the fourth cluster obtained samples 10, 11, and 12 (purple sweet potato samples). The linkage distance (illustrated on the abscissa axis) between the main clusters was nearly 28,000.

### 3.8. Artificial Neural Network Model

**Figure 11:**
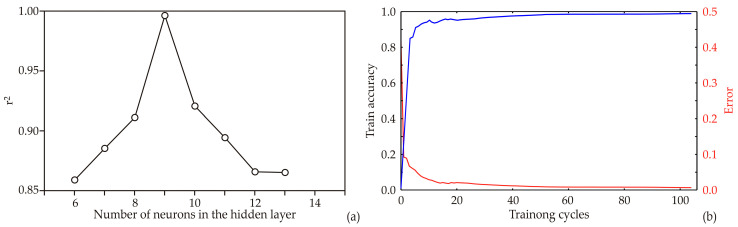
ANN calculation: (**a**) the dependence of the *r*^2^ value of the number of neurons in the hidden layer in the ANN model, (**b**) training accuracy (marked blue) and errors (marked red) per epoch.

This study involved the development of an ANN model, where its design and effectiveness hinged greatly on the initial assumptions made about matrix parameters such as biases and weight coefficients. These parameters hold significant sway in shaping the ANN to match experimental data precisely. Additionally, the quantity of neurons in the hidden layer can notably influence the model’s performance. To mitigate potential problems, each structure underwent 100,000 iterations to eliminate any chance correlation arising from initial assumptions and random weight setup. This careful method led the ANN model to reach its peak *r*^2^ value during training, notably with nine hidden neurons (refer to Figure 11a).

The ANN model underwent a 100-epoch training process, displaying its training outcomes in Figure 11b, explicitly focusing on the train accuracy and error (loss). The accuracy displayed a consistent rise with each training cycle until it reached a plateau roughly between the 30th and 50th epochs. Extending training beyond 50 epochs posed a notable risk of substantial overfitting. Hence, stopping at the 50th epoch proved sufficient to achieve high model accuracy without the threat of overfitting (see Figure 2).

Arguably, artificial neural network (ANN) models stand out as highly effective tools for tackling intricate design problems, mainly when dealing with complex parameter structures involving nonlinear relationships and connections [50]. Their adaptability, exceptional predictive accuracy, strong generalization capability, and resilience to noisy data make ANN implementation a promising approach for modeling complex systems [26]. The optimally designed neural network model, Table 5, demonstrated favorable generalization properties, facilitating the prediction of the observed parameters. These predictions were made based on the variety of sweet potatoes (white, pink, orange, and purple) and drying methods, including lyophilization, convective and osmotic drying. According to the ANN model calculations, the optimal number of neurons in the hidden layers for the ANN model was found to be nine, corresponding to network MLP 7-9-22. Furthermore, this model exhibited a high r-squared (*r*^2^) value, with *r*^2^ reaching 0.998, 0.998, and 0.998 during the training, testing, and validation, confirming the model’s validity.

The ANN model included multiple elements within their matrices, representing weighting and bias coefficients, as detailed in Supplement Tables S1 and S2.

### 3.9. ANN Model Validation

As presented in Table 6, the validation of the artificial neural network (ANN) model was conducted through both goodness-of-fit evaluation and residual analysis [51]. The results from the residual analysis revealed a random occurrence of residuals, underscoring the excellent fit of the model to the data. Skewness parameters were employed to assess the deviation of the distribution from normal symmetry. The calculated skewness value, approaching zero, indicated an asymmetrical distribution of the variables. Additionally, the kurtosis parameter, which gauges the “peakedness” of a variable’s distribution, was remarkably close to zero, signifying a normal distribution pattern.

### 3.10. Yoon’s Interpretation Method

Yoon’s interpretation method involved a sensitivity analysis that delved into the impact of various drying methods (such as convective drying, lyophilization, and osmotic dehydration) and the choice of sweet potato variety on the output variables.

**Figure 12:**
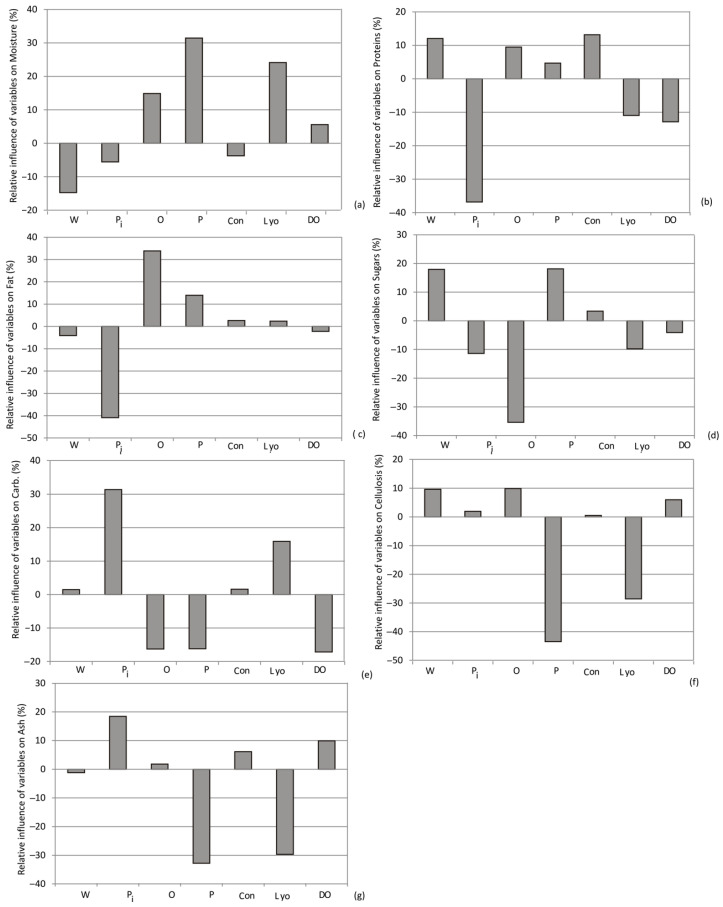
The relative importance of sweet potato variety and drying method combination on (**a**) moisture, (**b**) proteins, (**c**) fat, (**d**) sugars, (**e**) total carbon-hydrates, (**f**) cellulose, and (**g**) ash content. W, P, O, Pi—white, purple, orange, and pink sweet potato variety, respectively; Con, Lyo, and DO—convective drying, lyophilization, and osmotic dehydration (drying methods).

**Figure 13:**
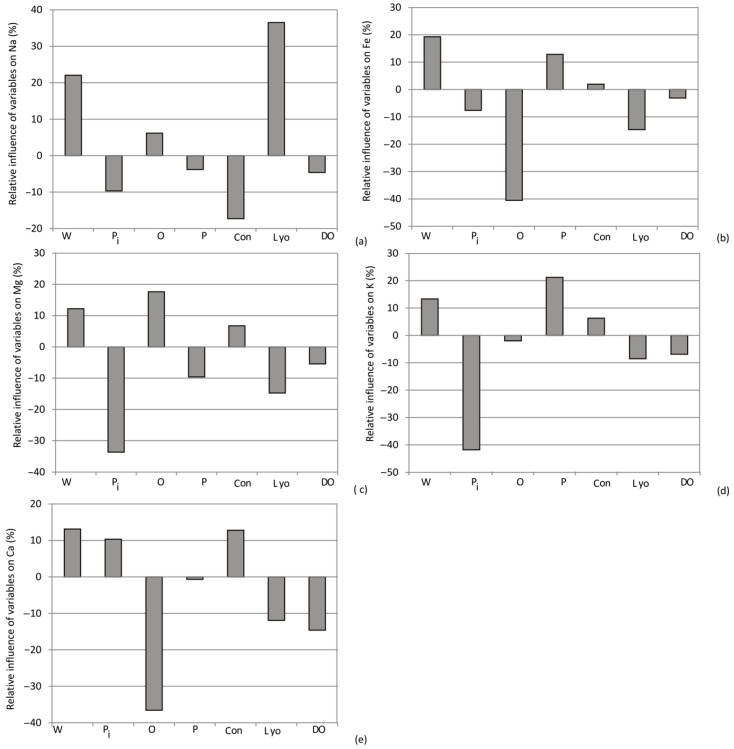
The relative importance of sweet potato variety and drying method combination on (**a**) Na, (**b**) Fe, (**c**) Mg, (**d**) K, and (**e**) Ca content (W, P, O, Pi—white, purple, orange, and pink sweet potato variety, respectively; Con, Lyo, and DO—convective drying, lyophilization, and osmotic dehydration (drying methods)).

**Figure 14:**
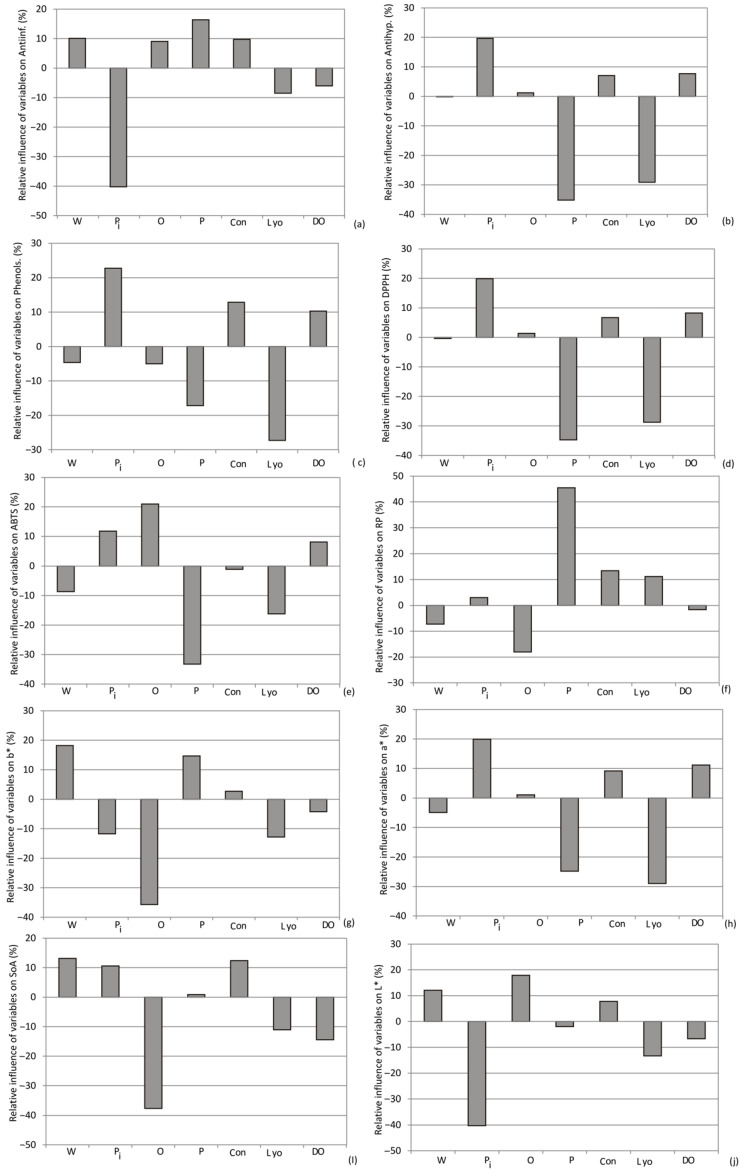
The relative importance of the type of sweet potato and the selection drying method on: (**a**) Phenols, (**b**) DPPH, (**c**) ABTS, (**d**) RP, (**e**) SoA, (**f**) Antinfl., (**g**) Antihyp., (**h**) a*, (**i**) b* and (**j**) L (W, P, O, Pi—white, purple, orange, and pink sweet potato variety, respectively; Con, Lyo, and DO—3.11. Drying Process Optimization.

As depicted in Figure 12, Figure 13 and Figure 14, the selection of purple sweet potato emerged as the most influential factor in evaluating phenols, DPPH, anti-hyperglycemic activity (%), a*, carbohydrates, and ash content, with relative importance values of +24%, +20%, +20%, +20%, +31%, and +19%, respectively. Additionally, among the drying methods, convective drying exhibited the highest influence on phenols (+12%), RP (+13%), SoA (12%), anti-inflammatory activity (10%), proteins (+12%), calcium (+11.8%), and magnesium content (+10%).

According to Yoon’s interpretation method, the impact of various drying methods and the choice of sweet potato variety on mineral composition are presented in Figure 13.

According to Yoon’s interpretation method, the impact of various drying methods and the choice of sweet potato variety on bioactive compounds and biological potentials are presented in Figure 14.

The optimization of the drying process parameters involved the application of the developed artificial neural network presented in Equation (5). One of the key objectives of this study was to maximize TPC, DPPH, ABTS, RP, SoA, anti-inflammatory activity, and anti-hyperglycemic activity simultaneously while minimizing moisture content after the drying process. This was achieved through the ANN model by varying the input parameters concurrently. The necessary mathematical computations were carried out using -Matlab’s multi-objective optimization of the outputs (MOO) calculations. The optimization constraints were applied within the experimental range of the variables. For the ANN model, the number of generations was 593. The population proportion was set to 100 for each input variable. Subsequent to the Pareto front, 79 points were identified for the model. During the drying process, the calculated maximum values were obtained for TPC, DPPH, ABTS, RP, SoA, anti-inflammatory activity, and anti-hyperglycemic activity of 1677.76 mg/100 g; 1500.56 μg TE/100 g; 10,083.37 μg TE/100 g; 3130.81 μg TE/100 g; 22,753.97 μg TE/100 g; 8.93% and 24.42%, respectively. The moisture content reached a minimum of 2.97%. Furthermore, the analysis revealed significant nutritional components in the sample. The protein content was measured at 12.6%, indicating a substantial protein presence. The fat content was notably low at 0.60%, while the sugar content was relatively high, reaching 17.29%. The sample also contained 3.97% cellulose, showcasing its dietary fiber content. Additionally, the ash content stood at 3.45%, indicating mineral presence. Carbohydrate content was determined to be 80.89%, suggesting a carbohydrate-rich composition. Regarding mineral composition, the sample exhibited notable levels of key elements. Potassium content was measured at 19,427.35 mg/kg, magnesium at 1241.44 mg/kg, calcium at 2841.53 mg/kg, iron at 21.86 mg/kg, and sodium at 516.69 mg/kg. Furthermore, the color analysis yielded specific coordinates: L* at 57.97, a* at 21.09, and b* at −9.36. These values provide valuable insights into the sample’s color characteristics. The optimal outcome was achieved for sample 10, which was the purple sweet potato variety. The drying process employed lyophilization. The study demonstrated that ANN can be effectively utilized as a modeling approach for predicting the drying of sweet potatoes. Moreover, the accuracy and precision of the developed ANN model suggest its applicability in postharvest management systems for sweet potatoes, serving as a valuable supporting tool in controlling the postharvest conservation process.

## 4. Conclusions

This study emphasizes the substantial impact of drying methods on sweet potato color properties, revealing distinct patterns for each variety. Additionally, this investigation offers insights into potential optimization strategies through advanced mathematical tools such as ANN modeling and multi-objective optimization. Chemical composition analysis underscores the effects of drying methods on nutritional components, while mineral composition analysis highlights the influence of both sweet potato variety and drying methods on key elements. Purple-fleshed sweet potatoes are identified as having the highest total phenolic content and antioxidant potential, emphasizing the link between color variations and bioactive properties. The developed artificial neural network (ANN) model effectively predicts parameters based on sweet potato variety and drying method, validated by high *r*^2^ values. The optimized sample, a purple sweet potato variety subjected to lyophilization, showcases significant nutritional components, including 12.6% protein, 0.60% fat, and 17.29% sugar. It also exhibits notable levels of key minerals. Color analysis provides specific coordinates, offering insights into the sample’s color characteristics. Overall, the study demonstrates the efficacy of the ANN model as a valuable tool for predicting sweet potato drying processes. The model’s accuracy and precision suggest its potential applicability in postharvest management systems, serving as a supportive tool in controlling the postharvest conservation process for sweet potatoes. With the outstanding potential of the presented ANN model, combined with a significant correlation of various drying methods and effects on the composition and bioactivities of sweet potato variety, the role of the ANN model could rapidly improve numerous food drying systems and their application at the industry level. Future research should focus on investigating the influence of drying techniques on different food nutrients in vegetables. By expanding the ANN database with additional experimental results, the prediction of the optimal drying technique for needed food products will be more effective, avoiding time-consuming and expensive sets of experiments to find an adequate solution.

## Figures and Tables

**Table 1:** Color characteristics of four different sweet potato varieties.

No.	Varieties of *I. batatas*	Drying Method	Coordinates		
			L* (Lightness)	a* (Red-Green)	b* (Yellow-Blue)
1	White	lyophilization	86.32 ± 0.06	−0.95 ± 0.03	16.99 ± 0.24
2		convective drying	80.65 ± 0.15	1.33 ± 0.11	18.72 ± 0.13
3		osmotic dehydration	65.36 ± 0.31	5.36 ± 0.02	23.17 ± 0.14
4	Pink	lyophilization	85.75 ± 0.09	−0.04 ± 0.01	14.69 ± 0.05
5		convective drying	85.75 ± 0.06	0.83 ± 0.02	17.74 ± 0.11
6		osmotic dehydration	63.38 ± 0.11	6.33 ± 0.03	25.02 ± 0.09
7	Orange	lyophilization	79.13 ± 0.06	17.66 ± 0.22	22.74 ± 0.30
8		convective drying	80.79 ± 0.08	7.32 ± 0.15	28.88 ± 0.29
9		osmotic dehydration	64.50 ± 0.10	18.04 ± 0.11	27.21 ± 0.13
10	Purple	lyophilization	57.97 ± 0.33	21.09 ± 0.21	−9.36 ± 0.01
11		convective drying	55.74 ± 0.15	16.61 ± 0.08	−0.01 ± 0.06
12		osmotic dehydration	55.26 ± 0.19	15.78 ± 0.08	−4.67 ± 0.04

**Table 2:** Chemical composition of four different sweet potato varieties.

*I. batatas* Variety	Drying Method	Chemical Composition Parameters						
		Moisture (%)	Proteins (% DW)	Fats (% DW)	Total Sugars (% DW)	Cellulose (% DW)	Ash (% DW)	Total Carbohyd-Rates (%DW)
White	lyophilization	5.12 ± 0.57	12.32 ± 0.63	0.72 ± 0.11	25.47 ± 3.43	4.53 ± 0.97	4.92 ± 0.37	77.85
	convective drying	6.03 ± 0.67	13.07 ± 0.67	0.57 ± 0.09	45.14 ± 6.01	5.47 ± 0.67	4.73 ± 0.36	76.71
	osmotic dehydration	7.1 ± 0.78	12.49 ± 0.65	0.55 ± 0.08	32.94 ± 4.35	4.15 ± 0.89	8.89 ± 0.67	72.52
Pink	lyophilization	3.2 ± 0.35	13.8 ± 0.71	0.61 ± 0.09	27.18 ± 3.73	4.55 ± 0.97	2.63 ± 0.2	80.31
	convective drying	4.61 ± 0.51	14.27 ± 0.74	0.68 ± 0.1	34.13 ± 4.62	4.7 ± 1.0	4.84 ± 0.37	76.51
	osmotic dehydration	9.05 ± 1.01	13.4 ± 0.69	0.57 ± 0.08	34.42 ± 4.42	3.78 ± 0.81	8.00 ± 0.6	70.92
Orange	lyophilization	3.48 ± 0.38	11.2 ± 0.58	0.75 ± 0.12	26.39 ± 3.61	3.5 ± 0.75	4.36 ± 0.33	80.77
	convective drying	3.88 ± 0.43	12.04 ± 0.62	0.83 ± 0.13	31.36 ± 4.27	4.22 ± 0.9	4.76 ± 0.36	79.17
	osmotic dehydration	7.93 ± 0.88	10.5 ± 0.54	0.92 ± 0.14	37.28 ± 4.87	4.39 ± 0.94	7.24 ± 0.55	74.89
Purple	lyophilization	2.97 ± 0.33	12.6 ± 0.65	0.60 ± 0.09	17.29 ± 2.38	3.97 ± 0.85	3.45 ± 0.26	80.89
	convective drying	2.27 ± 0.25	10.75 ± 0.55	0.54 ± 0.08	20.6 ± 2.85	4.33 ± 0.93	3.74 ± 0.28	83.04
	osmotic dehydration	5.53 ± 0.19	10.62 ± 0.45	0.42 ± 0.07	20.47 ± 2.79	3.85 ± 0.82	5.87 ± 0.44	80.05

**Table 3:** Mineral composition of four different sweet potato varieties.

*I. batatas* Varieties	Drying Method	Mineral Composition Parameters				
		K (mg/kg)	Mg (mg/kg)	Ca (mg/kg)	Fe (mg/kg)	Na (mg/kg)
White	lyophilization	26,140.70 ± 13.65	1009.14 ± 45.35	3179.92 ± 350.2	24.6 ± 1.56	415.92 ± 59.31
	convective drying	25,111.59 ± 2123.32	1358.6 ± 110.45	2211.47 ± 240.8	33.41 ± 3.15	52.96 ± 5.36
	osmotic dehydration	28,673.71 ± 2708.2	964.91 ± 109.91	2665.09 ± 210.7	72.91 ± 7.24	14,272.88 ± 1186.6
Pink	lyophilization	27,530.03 ± 2520.42	1660.47 ± 157.21	3565.12 ± 349.4	26.32 ± 1.92	560.74 ± 65.91
	convective drying	26,148.12 ± 2293.52	1116.89 ± 113.01	1975.157 ± 204.4	24.61 ± 2.79	869.513 ± 103.2
	osmotic dehydration	30,571.70 ± 3019.87	880.21 ± 106.35	1818.41 ± 180.2	71.95 ± 8.09	13,368.26 ± 1048.02
Orange	lyophilization	28,030.08 ± 2602.54	846.53 ± 101.13	1458.31 ± 124.7	17.37 ± 1.68	425.71 ± 35.22
	convective drying	27,759.82 ± 2558.16	899.41 ± 109.32	1840.84 ± 183.6	35.06 ± 2.41	605.2 ± 82.72
	osmotic dehydration	26,446.61 ± 2342.53	996.28 ± 94.33	1249.4 ± 142.5	35.23 ± 2.43	11,070.1 ± 1195.94
Purple	lyophilization	19,427.35 ± 1189.97	1241.44 ± 82.3	2841.53 ± 237.88	21.86 ± 1.37	516.69 ± 49.16
	convective drying	21,594.92 ± 1545.87	1025.13 ± 58.84	1786.42 ± 175.29	15.01 ± 1.31	516.44 ± 49.12
	osmotic dehydration	14,392.76 ± 10,771.55	720.05 ± 81.55	1483.48 ± 128.6	58.9 ± 5.08	6919.21 ± 700.02

**Table 4:** Bioactive compounds and biological potentials of four different *I. batatas*.

*I. batatas* Variety	Drying Method	Total Phenolic Content (mg/100 g DW)	Antioxidant Assays (μM TE/100 g DW)				Pharmacological Activities (%)	
			DPPH^●^	ABTS^●+^	RP	SoA	AIA	AHgA
White	lyophilization	191.44 ± 9.80	454.92 ± 25.52	988.46 ± 26.23	549.22 ± 29.08	18,793.31 ± 273.52	5.56 ± 0.2	8.06 ± 0.26
	convective drying	278.24 ± 16.17	687.53 ± 30.45	586.54 ± 32.48	1324.76 ± 29.03	21,305.91 ± 290.41	49.40 ± 1.29	61.56 ± 1.39
	osmotic dehydration	283.06 ± 17.35	619.05 ± 23.32	1855.91 ± 3.75	570.92 ± 17.21	18,651.15 ± 386.25	54.07 ± 1.83	3.37 ± 0.21
Pink	lyophilization	182.75 ± 10.12	297.73 ± 22.67	890.41 ± 3.12	451.67 ± 3.18	21,135.65 ± 281.68	50.62 ± 1.56	1.49 ± 0.04
	convective drying	207.45 ± 5.63	451.63 ± 27.58	1756.68 ± 68.48	1025.29 ± 2.92	18,221.36 ± 524.17	56.81 ± 1.9	40.10 ± 0.76
	osmotic dehydration	255.29 ± 7.16	500.41 ± 17.21	1720.31 ± 43.77	569.95 ± 24.16	13,714.78 ± 120.91	56.39 ± 1.46	4.18 ± 0.17
Orange	lyophilization	376.64 ± 27.89	680.66 ± 6.15	2025.80 ± 65.54	977.26 ± 17.00	21,367.07 ± 543.81	45.40 ± 1.13	6.51 ± 0.13
	convective drying	211.55 ± 11.78	449.92 ± 23.16	1857.67 ± 6.25	786.29 ± 12.75	17,619.65 ± 249.45	45.84 ± 0.45	44.40 ± 3.08
	osmotic dehydration	227.53 ± 17.13	381.78 ± 16.19	1584.72 ± 39.98	568.98 ± 4.54	8778.42 ± 431.31	58.72 ± 0.91	4.99 ± 0.48
Purple	lyophilization	1677.76 ± 61.23	1500.56 ± 35.63	10,083.37 ± 8.59	3130.81 ± 51.33	22,753.97 ± 50.17	8.93 ± 0.94	24.42 ± 0.50
	convective drying	1428.23 ± 82.94	1253.21 ± 31.09	7547.88 ± 89.21	2595.32 ± 31.17	22,529.16 ± 42.18	20.14 ± 0.68	23.04 ± 1.11
	osmotic dehydration	2006.81 ± 113.7	1172.29 ± 33.52	5316.85 ± 103.69	2544.64 ± 78.41	22,580.40 ± 52.56	5.94 ± 0.89	13.32 ± 1.44

TPC—total phenolic content; DPPH^●^—2,2-diphenyl-1-picrylhydrazyl; ABTS^●^^+^—2,2-azino-bis-3-ethyl benzothia-zoline-6-sulphonic acid; RP—reducing power; SoA—superoxide anion method; AIA—anti-inflammatory activity; AHgA—anti-hyperglycemic activity.

**Table 5:** Artificial neural network model summary (performance and errors) for training, testing, and validation cycles.

Network Name	Performance			Error			Hidden Activation	Output Activation
	Training	Testing	Validation	Training	Testing	Validation		
MLP 7-9-22	0.998	0.998	0.998	41,837.805	70,412.684	37,861.37	Tanh	Logistic
	Training Algorithm			Error Function				
	BFGS 865			SOS				

**Table 6:** The “goodness of fit” tests for the developed ANN model.

Tested Parameters	The “Goodness of fit” Validation Parameters											
	*χ* ^2^	*RMSE*	*MBE*	*MPE*	SSE	AARD	*r* ^2^	Skew	Kurt	Mean	StDev	Var
TPC (mg/100 g)	50.532	6.806	0.093	2.019	611.308	55.732	1.000	−0.931	0.699	0.102	7.455	55.573
DPPH^●^ (μg TE/100 g)	113.836	10.215	0.052	1.182	1.4 × 10^3^	68.509	0.999	−1.154	4.085	0.057	11.190	125.216
ABTS^●+^ (μg TE/100 g)	2.6 × 10^4^	154.896	−9.189	8.191	3.2 × 10^5^	1.0 × 10^3^	0.997	0.339	3.978	−10.024	169.354	2.9 × 10^4^
RP (μg TE/100 g)	1.4 × 10^3^	35.645	6.687	2.966	1.6 × 10^4^	245.060	0.998	1.597	2.623	7.295	38.290	1.5 × 10^3^
SoA (μg TE/100 g)	322.170	17.185	−1.485	0.065	3.9 × 10^3^	131.173	1.000	−0.226	1.647	−1.620	18.748	351.499
AIA (%)	0.001	0.031	−0.005	0.073	0.013	0.272	1.000	1.197	2.171	−0.005	0.034	0.001
AHgA (%)	0.257	0.486	0.026	5.426	3.104	3.576	0.999	−0.185	2.278	0.029	0.531	0.282
Moisture (%)	0.007	0.082	−0.011	1.509	0.086	0.597	0.998	−1.269	1.939	−0.012	0.089	0.008
Proteins (%)	0.006	0.072	0.020	0.228	0.063	0.303	0.997	3.264	10.749	0.022	0.076	0.006
Fat (%)	0.000	0.002	0.000	0.206	0.000	0.015	1.000	0.903	3.156	0.000	0.002	0.000
Sugars (%)	0.014	0.114	−0.009	0.332	0.170	0.950	1.000	−0.251	0.852	−0.010	0.124	0.015
Cellulose (%)	0.000	0.012	−0.002	0.202	0.002	0.099	0.999	−0.313	−0.761	−0.002	0.013	0.000
Ash (%)	0.005	0.070	0.000	0.845	0.064	0.490	0.998	1.818	5.159	0.000	0.076	0.006
Total carbs. (%)	0.119	0.330	0.005	0.236	1.436	2.066	0.991	0.406	3.863	0.005	0.361	0.131
K (mg/kg)	2.6 × 10^4^	155.136	3.440	0.387	3.2 × 10^5^	1.3 × 10^3^	0.997	−0.268	1.345	3.753	169.897	2.9 × 10^4^
Mg (mg/kg)	39.175	5.993	0.058	0.398	473.967	46.567	0.999	0.078	0.691	0.064	6.564	43.088
Ca (mg/kg)	110.732	10.075	−0.140	0.350	1.3 × 10^3^	78.373	1.000	−0.927	1.673	−0.152	11.035	121.780
Fe (mg/kg)	4.268	1.978	0.563	2.750	47.086	7.162	0.990	3.313	10.983	0.614	2.069	4.281
Na (mg/kg)	9.5 × 10^4^	294.347	130.12	22.393	9.0 × 10^5^	1.8 × 10^3^	0.998	1.810	2.436	141.944	286.016	8.2 × 10^4^
L*	0.016	0.122	−0.015	0.107	0.194	0.883	1.000	−1.338	1.442	−0.016	0.133	0.018
a*	0.226	0.455	0.076	−174.220	2.652	3.053	0.997	−0.304	2.278	0.083	0.491	0.241
b*	0.015	0.117	−0.022	−121.651	0.174	1.041	1.000	0.253	−0.401	−0.024	0.126	0.016

TPC—Total phenolics content; DPPH^●^—2,2-diphenyl-1-picrylhydrazyl; ABTS^●+^—2,2-azino-bis-3-ethyl benzothia-zoline-6-sulphonic acid; RP—reducing power; SoA—superoxide anion method; AIA—anti-inflammatory activity; AHgA—anti-hyperglycemic activity, *χ*^2^—reduced chi-square; *RMSE*—root mean square error; MBE—mean bias error; *MPE*—mean percentage error; SSE—total squared error; AARD—absolute average relative deviation; *r*^2^—coefficient of determination; Ske—skewedness; Kurt—kurtosis; StDev—standard deviation; Var—variance.

## Data Availability

Data are contained within the article. The data used to support the findings of this study can be made available by the corresponding author upon request.

## Supplementary Materials

The following supporting information can be downloaded at https://www.mdpi.com/article/10.3390/foods13010134/s1, Table S1. Elements of matrix *W*_1_ and vector *B*_1_ (presented in the bias row), Table S2. Elements of matrix *W*_2_ and vector *B*_2_ (presented in the bias column).
