# Supplementary material for: Unlocking the Potential of the ANN Optimization in Sweet Potato Varieties Drying Processes
Source: Foods. 2023 Dec 29;13(1):134. doi: 10.3390/foods13010134 (PMC10778433; doi:10.3390/foods13010134)
Supplement: Supplementary file 1 [file foods-13-00134-s001.zip › foods-2782492-supplementary.pdf]

Table S1. Elements of matrix W_1 and vector B_1 (presented in the bias row).

Tested Elements		Hidden Layer								
		1	2	3	4	5	6	7	8	9
I. *batatas* variety	White	-9.604	-9.209	-35.041	-3.771	-10.798	-5.382	-36.33	1.952	-8.451
	Pink	17.573	-8.665	3.626	-2.774	-10.479	-60.554	-4.741	2.003	-13.8
	Orange	19.137	27.568	5.917	-1.474	44.38	8.113	-2.628	1.683	1.428
	Purple	-12.55	-6.288	31.836	-0.167	-9.482	58.515	44.544	8.579	40.517
Drying method	lyophilization	0.145	6.557	-10.158	11.14	-1.34	-11.939	3.593	-18.618	-3.32
	convective drying	15.429	5.134	30.556	13.131	31.25	21.857	3.688	48.961	38.665
	osmotic dehydration	-1.076	-8.266	-14.044	-32.476	-16.335	-9.126	-6.562	-16.164	-15.724
	Bias	14.499	3.474	6.283	-8.262	13.578	0.714	0.86	14.3	19.736

Table S2. Elements of matrix W_2 and vector B_2 (presented in the bias column).

Tested Parameters	Hidden Layer									Bias
	1	2	3	4	5	6	7	8	9	
L*	-27.005	12.446	-21.34	35.893	51.947	-3.112	-15.006	-0.407	-60.651	26.218
a*	51.656	30.708	-26.193	-0.362	-42.442	62.428	22.871	-60.146	-15.575	-20.199
b*	-6.177	25.551	17.045	7.655	-32.208	-3.62	-23.391	0.35	6.254	5.621
TPC (mg/100g)	25.493	16.451	-30.392	0.509	-23.165	16.76	27.371	-11.572	1.742	-21.92
DPPH (µg TE/100g)	5.159	-4.512	-10.81	5.245	-2.219	56.132	4.818	-56.116	-21.246	23.193
ABTS (µg TE/100g)	-8.332	-8.491	-5.926	-3.724	14.569	2.145	9.588	-0.898	-6.457	6.732
RP (µg TE/100g)	19.9	15.365	-24.135	0.446	-20.057	-13.744	23.454	17.072	14.735	-31.656
SoA (µg TE/100g)	3.75	-2.315	-13.313	74.601	-72.897	1.537	-14.484	-12.565	34.005	2.145
AIA (%)	-3.118	57.086	-51.829	33.408	5.712	-38.191	14.58	39.483	-37.248	-17.222
AHga (%)	-3.225	-7.905	-9.835	4.191	4.711	40.798	6.036	-43.83	-15.792	23.221

**Moisture (%)**	16.014	2.294	2.949	-1.594	-6.011	0.366	-2.774	-0.659	2.982	-15.706
**Proteins (%)**	30.348	-5.67	-48.229	94.818	30.955	-0.613	-12.721	-10.888	-54.216	-24.022
**Fat (%)**	4.584	3.113	1.885	0.603	1.571	0.019	-2.654	-0.564	-3.788	-5.504
**Sugars (%)**	-1.546	17.985	11.082	5.562	-23.954	-2.874	-16.386	0.332	6.07	0.701
**Cellulosic (%)**	-31.381	-19.069	-10.719	-4.503	31.342	0.933	12.429	-2.848	-10.563	31.189
**Ash (%)**	8.185	3.517	0.332	1.505	-8.407	37.72	-3.376	-39.258	-14.559	12.656
**Total carbs. (%)**	-1.92	0.188	-0.135	0.994	-0.505	-0.141	0.038	0.189	2.122	-1
**K (mg/kg)**	1.226	5.14	2.582	2.406	-4.319	-0.833	-4.651	0.2	-1.04	-1.393
**Mg (mg/kg)**	-26.988	-6.916	-18.37	12.265	39.712	-1.126	4.092	0.746	-29.558	25.047
**Ca (mg/kg)**	0.046	-14.03	-20.801	85.667	-70.203	2.498	-10.097	-16.036	34.662	7.434
**Fe (mg/kg)**	-33.431	88.848	62.824	25.03	-116.925	-13.966	-83.943	0.843	25.591	31.974
**Na (mg/kg)**	-10.318	-4.439	-32.354	-11.697	16.094	-2.477	10.358	49.951	-2.91	-15.951

TPC-Total phenolics content; DPPH•-2,2-diphenyl-1-picrylhydrazyl; ABTS•+-2,2-azino-bis-3-ethyl benzothia-zoline-6-sulphonic acid; RP - reducing power; SoA - superoxide anion method; AIA - anti-inflammatory activity; AHgA - antihyperglycemic activity
